# Online digital health and informatics education for undergraduate nursing students in China: impacts and recommendations

**DOI:** 10.1186/s12909-024-05785-5

**Published:** 2024-07-26

**Authors:** Hongxia Shen, Chong Chen, Sijing Yan, Cynthia Hallensleben, Rianne van der Kleij, Minyi Li, Huohuo Dai, Niels Chavannes, Ying Zhou

**Affiliations:** 1 School of Nursing, Guangzhou Medical University, 195# Dongfeng West Road, Guangzhou, Guangdong 510182 China; 2 Department of Public Health and Primary Care, Leiden University Medical Centre, Leiden, Netherlands; 3 National eHealth Living Lab, Leiden University Medical Centre, Leiden, Netherlands

**Keywords:** Digital health, Informatics, Chinese nursing students, Education, Competency, Knowledge

## Abstract

**Background:**

Digital health plays a vital role in healthcare services. Governments in many countries, including China, are increasingly advocating for the appropriate use of digital technologies to address significant health system challenges. It is crucial to incorporate digital health education into the curriculum for future nurses to adapt to the changes in the digital medical system. This study aimed to evaluate the impact of an online Digital Health and Informatics Course in China on the knowledge and comprehension of key digital health and informatics topics, self-assessment of nursing informatics competencies, and satisfaction among undergraduate nursing students. The findings of this study provide recommendations for the design and implementation of future digital health education.

**Methods:**

This study employed a one-group, quasi-experimental mixed-methods design with pre- and post-assessments. The participants received digital health and informatics education through six three-hour online sessions in six interactive days, with online self-learning materials in between. An online quiz and focus group discussions pre- and post the course were designed to evaluate the knowledge and comprehension of key digital health and informatics topics. Also, a validated Chinese version of the Self-assessment of Nursing Informatics Competencies Scale was conducted pre- and post-course to assess self-assessment of nursing informatics competencies. Additionally, all students were invited to participate in an online survey with a performance-focused course evaluation form as well as focus group discussions to gather their feedback on the learning experience and their evaluations of the course.

**Results:**

A total of 24 undergraduate nursing students were enrolled in the course. All students completed all sessions of this course, resulting in an attendance rate of 100%. Additionally, all students completed both pre- and post-assessments. In terms of the knowledge and comprehension of key digital health and informatics topics, scores of the quiz on knowledge assessment improved from the pre-test [mean pretest score: 78.33 (SD 6.005)] to the post-test [mean post-test score: 83.17 (SD 4.86)] upon completion of the course (*P* < 0.001). Also, students acknowledged that the course enhanced their knowledge and comprehension of informatics and digital health, the benefits of (nursing) informatics in clinical practice, and the role of health care professionals in informatics and digital health. In terms of self-assessment of nursing informatics competencies, scores on nursing informatics attitudes demonstrated significant improvement *(P* < 0.001). Furthermore, students reported high satisfaction with various aspects of this course, including the opportunity to explore broad horizons in informatics for future careers, engaging in group discussions, and analyzing case studies on the use of informatics and digital health in clinical practice.

**Conclusions:**

This Online Digital Health and Informatics education effectively improved undergraduate nursing students’ knowledge and comprehension of the key digital health and informatics topics, nursing informatics attitudes in the self-assessment of nursing informatics competency with high levels of satisfaction. In order to ensure that future education in digital health and informatics for nursing students is in line with the technological advancements in clinical settings, it is necessary to foster collaboration between medical school training and clinical practice. This collaboration should involve the use of clinical examples to illustrate advanced digital health applications and the inclusion of practical exercises on the use of digital health technology in clinical settings.

**Supplementary Information:**

The online version contains supplementary material available at 10.1186/s12909-024-05785-5.

## Background

Digital health is defined by the World Health Organization (WHO) as the field of knowledge and practice associated with the development and use of digital technologies to improve health. Developments such as digitally mediated diagnosis and treatment, cloud computing, machine learning, artificial intelligence, block-chain, telehealth, and consumer-facing mobile health applications have enhanced the delivery of care for individuals across the spectrum of health promotion and disease prevention, diagnosis, treatment, and rehabilitation [1, 2]. Digital health solutions are also recognized for their benefits in nursing practice, including integrating data records across various databases, providing electronic decision support and resources, and developing digital devices that facilitate remote monitoring and individuals’ positive behavior change [3–5]. Especially during the coronavirus disease (COVID-19) pandemic, digital health applications have been noted as an innovative health solution that improves continued healthcare accessibility and streamlines public health action to stop the rapid spread of the crisis [6, 7].

Governments in the United States, India, Tanzania, Ethiopia, and other countries have developed national digital health strategies, which outline a shared vision for addressing health priorities through the coordinated and strategic use of interoperable digital technologies [8–10]. As the largest developing country, policymakers and healthcare experts in China have launched the national health strategy ‘Healthy China 2030’ [11]. This strategy recognizes digital health technology as an essential pillar to enhance disease self-management, as well as improving the accessibility and cost-effectiveness of care in (rural) China- where over 558 million people have access to mobile phones. According to the correspondence from the National Health Commission of China, as of August 2022, more than 1700 internet hospitals have been established nationwide [12]. In order to promote the appropriate use of digital technologies and therefore help address key health system challenges in general and in China in specific, it is important to enhance individuals’ understanding and use of digitally enabled approaches to care. This will ultimately lead to improved quality of care, better health outcomes and reduced medical costs.

Currently, numerous digital health technologies remain in the pilot stage and have not yet demonstrated their effectiveness or been successfully implemented on a larger scale in a real-world setting. The limited knowledge and skills of healthcare professionals (HCPs) in utilizing new technologies and concerns regarding privacy, and security quality are significant obstacles to the adoption of digital health in clinical practice [13]. The Global Digital Health Strategy 2020–2025 of WHO emphasizes the importance of incorporating specific actions to ensure that all health professionals and allied workers, at all levels of formal education and informal training, receive education and training on digital health [14]. Developing high levels of digital health informatics competencies among health professional students will facilitate their understanding of the essential requirements for successful implementation of digital health [15]. Therefore, there is an urgent need to develop courses on digital health in medical schools to educate future HCPs on integrating digital health technological innovations and preparing them to adapt to future changes in the digital medical system within their workforce.

At present, many medical schools and research institutions worldwide have incorporated the digital health education into their curricula for the next generation of HCPs. For instance, digital health education programs were designed and implemented as mandatory or elective courses for bachelor or master health professional students at the University of Maryland-Baltimore, Vanderbilt University, Duke University, New York University, the University of Pittsburgh [16, 17]. These programs cover a range of topics, including an introduction to health informatics, system design, implementation, evaluation, and policy and ethics in digital health.

While there has been an increasing amount of research on digital health education within the medical school curriculum in Western countries, a systematic review has shown that the literature on digital health courses often lacks comprehensive evaluation, and more evaluation and implementation research is recommended in low and middle-income countries [17]. Also, there is a lack of literature on this topic in the Chinese context. Health professional students in China have expressed a need for digital health knowledge and skills in their medical curriculum [18].

Nurses, as the largest group of HCPs, play a crucial role in the digital health transformation. It is important to enhance the readiness and education of the nursing workforce in digital health to provide effective, safe, and efficient patient care with the support of existing and emerging digital health technologies. Therefore, to prepare future nurses to meaningfully contribute to the design, development, implementation, and evaluation of digital health technologies in China, the present study aimed to evaluate the impact of an online Digital Health and Informatics Course in China on undergraduate nursing students’ knowledge and comprehension of key digital health and informatics topics, self-assessment of nursing informatics competencies, and satisfaction. Based on the actual experiences of nursing students, our study will also provide recommendations for medical educators, medical universities, and health institutions to improve the design and implementation of digital health education in China and beyond.

## Methods

### Study design and participant

This study employed a one-group, quasi-experimental mixed-methods design with pre- and post-assessments. The study focused on an online Digital Health and Informatics Course for undergraduate nursing students in China. All students who enrolled in the course at our university, a medical university located in Guangzhou, southern China, were invited to participate in the study. To be eligible, students had to agree to participate and successfully complete the six-week course. Students who expressed disinterest or were already engaged in other digital health learning or programs were excluded from the study.

### Course details

#### Development of course

A multidisciplinary team of experts in digital health, informatics, and the medical field was established at a medical university in Guangzhou, southern China. The team consisted of three nurses, two doctors, two health informatics specialists, and a teacher with extensive experience in designing and conducting medical courses for nursing students. The team held monthly meetings to discuss the development, implementation, and evaluation planning of the course.

The course was developed in three stages. Firstly, a comprehensive list of digital health and informatics areas was generated based on previous education in this field [16, 17]. This list was used to define the most relevant topics for nursing students. Secondly, the team of eight experts reviewed the objectives, learning activities, and assessment tools for the course. Based on this review, a proposed course with five core topics was devised, which all eight experts agreed upon. Thirdly, the course procedure was enhanced by incorporating multimedia learning materials such as illustrations, photos, animations, and videos. This was in accordance with the Multimedia Learning Theory [19]. Through group meetings and discussions, the multidisciplinary team reached a consensus on the final course module and procedure.

#### Digital health and nursing innovation topics

The course was named as the Digital Health and Informatics course and was held at a medical university in Guangzhou, China, for eleven days in six weeks. Details of the overview and content of the course are shown in additional file 1. The content of this course focused on five key topics: digital health informatics, nursing informatics, emerging technologies for eHealth solutions, patient data security and privacy, and eHealth. The timetable and focus of each topic are shown in Table 1.

In the final week of the course, students were assigned group work-based learning activities to present a critical reflection on the following three questions:

1. Given the recent emergence and certification of health informatics professionals, will there continue to be a role for nurses in informatics within the next decade?
2. Should there be a specific professional designation for nurses with informatics expertise? What will the role of the Informatics Nurse look like in the future?
3. In the face of the evolving sophistication of technology, will there still be a need for nurses with informatics expertise?

During the group work, students were encouraged to read materials such as scientific papers and textbooks related to digital health and nursing informatics. Following each group’s presentation, the teachers provided feedback on students’ performance and on the material they were presenting, thus enabling them to review their strengths, areas that needed improvement, their development and learning, and to reconsider their learning processes.

**Table 1:** Topics of Digital Health and Informatics course

Week	Time duration	Learning objectives	Details	Procedure
Week 1	3 h	• Able to determine what is health informatics • Able to formulate a type of Health Informatics • Knows the elements that health informatics should consist of • Knows the formalities of digital health informatics	**Lecture 1: Digital Health Informatics** • Definition of Health Informatics • Health Information Systems • Standardization and Interoperability • Tutorial 1	• The teacher introduced the topics related to basic information about information management, digital health, health informatics, and forces driving information technology (e.g., patient safety, nursing shortage, genomics, demands for cost-efficient, quality care) • Course structure ­ Lectures ­ A written tutorial formulated by 15 multiple-choice questions
Week 2	3 h	• Able to explain the definition and importance of nursing informatics • Able to explain the difference between nursing informatics and other fields • Able to describe the role of nurses in digital health management	**Lecture 2: Nursing Informatics** • Definition of Nursing Informatics • Emerging eHealth Agenda • Nursing Early Role in Medical Informatics • Nursing Informatics: Progression of a Specialty • Impact of Nursing Informatics on Nursing • Nursing Informatics Competencies • Tutorial 2	• The teacher introduced the topics of Nursing Informatics definition, scope of systems used in Nursing, specialty area for practice, and its application. • Course structure ­ Lectures ­ A written tutorial covering three discussion questions and 10 multiple-choice questions
Week 3	3 h	• Able to elaborate on the use of emerging eHealth technologies in clinical practice and give examples • Able to explain the difference between complex and complicated systems. • Able to describe the strategies used in eHealth technologies for simple, complex, or complicated interventions. • Able to describe the eHealth solution used on different levels	**Lecture 3: Emerging Technologies for eHealth Solutions** • Service Oriented Architecture (SOA) and Web Services • Cloud Computing • Wireless Technologies and the Mobile Internet • Health Informatics Standards	• The teacher introduced the topics on a range of technologies used in clinical practice. • Course structure - Lectures - Workshops
Week 4	3 h	• Able to describe the principles of the use of digital health technologies that need to be adhered to in the clinical practice • Able to explain, recognize, and give examples of privacy principles in clinical practice • Able to explain the state of the art and the gaps in knowledge and practice of patient data security and privacy	**Lecture 4: Patient Data Security and Privacy** • Why patient privacy matters • Definitions • What constitutes personal health information? • What determines the sensitivity of personal health information? • Privacy Principles • Information Security Principles • The Role of Nurses in Maintaining the Privacy and Security of Personal Health Information	• The teacher introduced the importance of patient privacy and security • Course structure - Lectures - The workshops were highly interactive and unstructured, with an emphasis on hands-on practice time and group discussion on formulation of patient privacy and security questions, critically appraising the literature, and applications to patient scenarios.
Week 5	3 h	• Able to understand how eHealth can support self-management in daily life • Able to critically appraise eHealth applications in terms of content, impact, implementation, advantages/disadvantages, and evidence	**Lecture 5: eHealth: The application of Technology and Data in Modern Health and Social Care Services** • Electronic Medical Records • Telehealth • mHealth • Cloud Computing • Tutorial 3	• The teacher introduced the definition of eHealth its importance, and key factors for the successful implementation of the eHealth system. • Course structure ­ Lectures ­ Small group tutorials ­ Task-based assignments ­ Internet-based instruction ­ Tutorials covering description of nursing informatics nurses’ work and sharing experience
Week 6	3 h	• Able to gain insight into the role of HCPs in informatics, a specific professional designation for HCPs with informatics expertise, and the specific role of informatics nurses	• Team Project • Presentation • Concluding • Remarks • Closing Session	• Students present the reflection on the role of HCPs in informatics, a specific professional designation for HCPs with informatics expertise, and the specific role of informatics nurses in small groups. The integration of knowledge and skills was encouraged in formative evaluations spaced throughout the class. • Course structure -Presentation

#### Teaching members

For the Digital Health and Informatics course, teachers were eligible if they had extensive experience in digital health and informatics, software engineering, information management, and knowledge management. They were also eligible if they had previous teaching experience in software engineering and enterprise systems development, or if they had conducted wide-ranging research in the areas of information sharing, healthcare informatics, artificial intelligence, machine learning, and digital health.

In this course, all lectures were delivered by two teachers, all of whom hold a Doctoral degree and have a proven academic or professional background in the fields of software engineering, artificial intelligence, and information systems, or digital health research, nursing, public health, and implementation science. Additionally, both teachers have obtained Teacher Qualification Certificates, which demonstrate that they possess the basic teaching skills necessary to perform educational and teaching activities in higher education. Furthermore, both teachers have in-depth knowledge of the course content, aligning with the course’s subject expertise. Moreover, they have more than three years of teaching experience and were extensively involved in digital health course planning and education administration. As a result, they are capable of delivering engaging online lessons, promoting interactive student online participation, and maintaining open discussions between students and teachers.

#### Course procedure

The course commenced on July 23, 2022. The students who enrolled in the course were sent a link via email. In order to take part in the study, they were required to provide informed consent and complete the electronic baseline questionnaires. Following each lesson, students were granted access to the course through various modalities, including offline and online access to downloadable lesson videos for offline viewing on their electronic devices. Participants had the flexibility to watch the modules in their preferred order. The course structure for each topic is outlined in Table 1.

#### Evaluation

The assessment of the impacts of the course was conducted using a mixed methods approach to evaluate (1) knowledge and comprehension of the key digital health and informatics topics, (2) the self-assessment of nursing informatics competencies, and (3) the students’ satisfaction with the Digital Health and Informatics course. Table 2 summarizes the below-described outcome and outcome measurements.

**Table 2:** Field methods, outcomes and measurements used in the mixed-method study

Outcome	Outcome measurement	Methods		Pre- Course	Post- Course
		Survey or quiz	Focus group discussion		
Knowledge and comprehension of the key digital health and informatics topics	• An online quiz pre-and post the course with a total score of 100 points • Focus group discussions regarding comprehension of informatics and digital health topics	X	X	X	X
Self-assessment of nursing informatics competencies	• A validated Chinese version of the Self-assessment of Nursing Informatics Competencies Scale	X		X	X
Students’ satisfaction with the Digital Health and Informatics course	• An online survey with a performance-focused course evaluation form • Focus group discussions regarding evaluations and suggestions of the course	X	X		X

### Research materials

#### The demographic questionnaire

The questionnaire was used to collect students’ demographic data, including age, gender, year of bachelor study, and experience with nursing informatics use.

#### Knowledge and comprehension of key digital health and informatics topics

To evaluate the knowledge and comprehension of key digital health and informatics topics, an online quiz with a total score of 100 points and focus group discussions pre-and post the course were designed.

The quiz consisted of a total of 25 choice questions that were developed by the multidisciplinary team of experts. These questions were considered important learning topics and were based on relevant lecture materials. Before finalizing the quiz, a pilot version was tested by ten nurses who had graduated from the same medical university within one year and had experience with digital health learning. The pilot test aimed to improve the content, length, and understandability of the quiz. The final version of the quiz focused on eliciting students’ knowledge and comprehension of digital health, health informatics, and nursing informatics including definitions, nursing’s early role, and nursing informatics competencies (see additional file 2).

In addition to the quiz, all students were invited to participate in focus group discussions pre- and post-course. The focus group topic lists were developed based on examples from similar studies and research team discussions. The focus group discussion questions aimed to assess participants’ knowledge and comprehension on informatics and digital health such as definitions and emerging technologies. The discussion also explored the importance of informatics and digital health as well as the role of nurses in nursing informatics and digital health implementation (See additional file 3).

#### Self-assessment of nursing informatics competencies

All students were invited to participate in an online nursing informatics competency survey pre- and post-course. According to previous literature [20], nursing informatics competencies include not only computer-related skills, but also the knowledge and attitudes needed by nurses to complete specific informatics tasks. The online survey consisted of two parts (See additional file 4).

• Part one provided instructions for completing the survey.

• Part two included a validated Chinese version of the Self-assessment of Nursing Informatics Competencies Scale (SANICS) [21] developed by Yoon [22]. The scale consisted of a total of 28 items, covering three domains: computer technology, information technology, and information knowledge. The Cronbach’s alpha of the Chinese version of SANICS was 0.931 [21]. Five-point Likert-type criteria was applied (1 = not competent; 2 = somewhat competent; 3 = competent; 4 = proficient; 5 = expert), with a higher total score indicating a higher level of nursing informatics competency. The Chinese version of SANICS items were categorized into 5 sub-scales: role of clinical informatics (Factor 1; items 1–5), basic computer knowledge and skills (Factor 2; 6–16), applied computer skills (Factor 3; 17–20), wireless device skills (Factor 4; 21–24), and nursing informatics attitudes (Factor 5; 25–28). The five domains and examples of items are presented in Table 3.

**Table 3:** Domains in the self-assessment of nursing Informatics competencies Scale

Domain	Explanation	Examples of scale items
Role of clinical informatics	The practice that nurses implemented in the clinical informatics	Promote the integrity of and access to information to include but not limited to confidentiality, legal, ethical, and security issues
Basic computer knowledge and skills	Techniques and tools in systems analysis and project management	Use database management program to develop a simple database and/or table
Applied computer skills	Use of computer hardware and software	Perform basic trouble-shooting in applications
Wireless device skills	Wireless device to locate resources and enter data	Use wireless device (PDA or cellular telephone) to enter data
Nursing informatics attitudes	Evaluations on nursing informatics	Recognize that internet + plus will become more comment

#### Satisfaction with the Digital Health and Informatics course

Following the implementation of the course, all students were invited to join an online survey using a performance-focused course evaluation form (See additional file 5). The survey aimed to gather feedback on students’ learning experience and obtain specific comments regarding the course. Also, students were invited to take part in focus group discussions on the course evaluation. The focus group discussion questions were as follows:

- “What do you like about the course?”
- “What do you dislike about the course?”
- “Do you have any suggestions on the future improvement of the course?”

### Data collection

#### Quiz and survey

Prior to the study, participants were provided with information regarding the purpose of the study. They were asked to complete web-based questionnaires in the form of an online quiz, SANICS surveys, and course evaluation forms. The surveys were conducted between June and July 2022. A link containing a password to access the private survey questionnaires was sent to each student’s individual email inbox. Participants were informed that their participation in the study was voluntary and that choosing not to participate would not affect their learning or assessments. They were assured that they could withdraw from the study at any time without any negative consequences or impact on their academic grades. Furthermore, their privacy and confidentiality would be protected, and all participants provided written consent to participate. Participation in the online poll was also voluntary and anonymous.

#### Focus group discussions

A total of five pre- and post-course focus group discussions were conducted with all students to explore their knowledge and comprehension of key topics in digital health and informatics, as well as their satisfaction with the course. The face-to-face focus group discussions were conducted by one researcher (HS, PhD, female). The interviewer had received training and possessed extensive experience in qualitative research. Each focus group discussion lasted approximately 50–60 min and was recorded with the participants’ consent. The recordings were later transcribed and used as textual data.

### Data analysis

For the quantitative data, survey data were exported from SPSS version 23 (IBM, Armonk, NY, USA) for analysis. After data cleaning, frequency descriptive statistics were utilized for categorical variables. Descriptive statistics such as the mean, standard deviation, median, and range of linear variables were calculated, along with frequencies and percentages of categorical variables. We compared the difference of the SANICS scores pre- and post-course using paired *t-test* analysis. *P*-values < 0.05 was considered statistically significant.

For the focus group discussion data, transcripts were imported into Atlas.ti for Windows version 7.5.18 (Scientific Software development, Berlin). Qualitative content analysis was performed inductively using the following steps: (1) open coding, (2) categorization, and (3) theming. Rigor was enhanced by repeatedly reading the transcripts, keeping a record of the analytic decision trail, and through crystallization with multiple researchers engaging in discussions of evolving categories and emergent themes. In terms of students’ knowledge and comprehension of key digital health and informatics topics, related quotations were compared to identify the changes in the same themes extracted pre- and post-course. For instance, the theme of emerging digital health technologies was extracted from pre- and post-course focus group discussions. We will compare the differences in relevant quotations, such as whether students mentioned more types of technology after the course.

Additionally, based on the focus group discussion data and responses to two open-ended questions in the course evaluation form provided by students, we analyzed their evaluations and suggestions regarding the course. Data saturation was achieved as being the point at which no new or relevant information could be identified through the iterative, preliminary analysis of the data [23]. After the first two focus group discussions, a preliminary analysis using the proposed codes was performed, and a data saturation grid [23] was developed to determine if saturation was reached. The data saturation grid consists of a report of the occurrence of themes and codes (displayed in rows) during each focus group (displayed in columns) in a tabular format. In the grid, saturation is considered reached when the grid column for the current focus group indicates no new information emerged for that particular theme or code. We found that in the fifth focus group discussion, data saturation on all themes and codes was achieved (data saturation table included as additional file 6).

### Ethics and consent

This study was assessed and approved by The University Ethics Committee of Guangzhou Medical University (Reference Code: L202303012). All methods were carried out per relevant guidelines and regulations. Informed consent was obtained from all participants.

## Results

### Demographic characteristics of students

A total of 24 undergraduate nursing students were enrolled in the course. All students completed all sessions of this course (attendance rate 100%) and pre- and post-assessments. Most participants (83.33%; *n* = 20) were in the 19 to 20-year age category. Also, 22 students (91.7%) were in the first or second year of their bachelor study. Additionally, students’ experience with nursing informatics was limited before launching the digital health and nursing informatics course, with only 41.7% of students having prior experience with nursing informatics system use.

### Impact of the Digital Health and Informatics course

#### Knowledge and comprehension of key digital health and informatics topics

Scores of the quiz on knowledge assessment improved from the pre-test [mean pretest score: 78.33 (SD 6.005) to the post-test [mean post-test score: 83.17 (SD 4.86)] upon completion of the course (*P* < 0.001).

Furthermore, when analyzing the data from focus group discussions, three key themes emerged regarding nursing students’ knowledge and comprehension of key digital health and informatics topics before and after the course. Overall, the students acknowledged that the course improved their understanding of informatics and digital health, the benefits of (nursing) informatics in clinical practice, and the role of HCPs in informatics and digital health. For instance, after the course, the theme of ‘Understanding of informatics and digital health’ was formulated based on constructs that emphasized the application of information management and analytical abilities, more guiding principles of nursing informatics and digital health use, and more emerging technologies of digital health compared with pre-course. Also, after the course, students highlighted detailed technologies for improving clinical practice. In addition, students noted the different types of informatics roles held by HCPs working in the field of informatics and the critical roles they play after the course. The main constructs frequently mentioned by students were summarized from the interview transcripts before and after the course, which were translated into English from colloquial Chinese, supporting these findings (Table 4).

**Table 4:** An overview of the main constructs of themes and subthemes mentioned by students pre-and post-course

Themes and subthemes	Pre-course Main constructs mentioned	Post-course Main constructs mentioned
**Theme 1 Understanding of digital health and informatics**		
Definition of digital health and (nursing) informatics	- A discipline that includes nursing practice assessment based on nursing information data.” - The processing of collecting nursing or patient data and information - The application of information technology to assist nursing care - An interdisciplinary practice of nursing and informatics	- A science with multiple information management and analytical abilities in addition to information technologies - A cross-discipline to improve nursing and requires a change in thinking and competence - The application in nursing research, nursing science, nursing research, nursing education and nursing management. - The urgent need to use technology in medical settings to ensure patient safety - Provides needed standardized nursing language
Guiding principles of informatics practice and use of digital health	- Patient privacy - Patients’ willingness to use digital health - Accurate data entry	- Patient privacy and safety - Efficient, timely, and accurate data collection and entry - Data security - Protecting confidentiality of personal information - Confidentiality - Ease of use of digital health
Emerging technologies of digital health and informatics	- Electronic health records - Personal digital assistant - Tablet for patient data collection - Internet-based outpatient clinic reservation - Mobile phone-based patient discharge follow-up and health education	- Wireless technologies - Telemedicine - Electronic health records - Personal digital assistant - Tablet for patient data collection - Internet-based outpatient clinic reservation - Mobile phone-based patient discharge follow-up and health education
**Theme 2 Benefits of digital health and informatics in nursing practice**	- Improving the efficiency of care with online documentation - Facilitate information integration, access, and retention with monitoring devices recording patient measurements - Ensure the accuracy of patient information and care with automatic reminder in documentation and wearable devices for data collection - Self-monitoring by patients helped health care professionals to provide accurate medical advice, based on the changes in parameters or symptoms tracked	- Monitoring devices that record vital signs and other measurements directly into the client record - Automatic reminder with nursing documentation - Reminders and prompts that appear during documentation to ensure comprehensive charting. - Quick access to computer-archived patient data from previous encounters - Automated staff scheduling for nursing administration - The ability to find trends in aggregate data, which is data derived from large population groups. - Use of the Internet for obtaining data collection tools and conducting research. - Communication with healthcare providers via e-mail and instant messaging. - Remote monitoring and other telehealth services.
**Theme 3 The role of health care professionals in informatics**	- Provide feedback on care information needs to technicians - Participate in the selection, design, implementation, and evaluation of information systems - Act as a leader and be able to integrate innovative and informatics concepts into their area of expertise - Generalize or promote knowledge or application of skills in information systems to others	- The different roles of the Beginning Nurse, the Experienced Nurse, and The Informatics Nurse, and The Informatics Nurse Specialist - Implementer: • Use relevant information and knowledge to support the delivery of evidence-informed patient care. • Use information and communications technology in accordance with professional and regulatory standards and workplace policies (e.g., protection of health information, privacy, and security) - Informatics systems development - Informatics systems use and evaluation - Act as educators to deliver informatics knowledge to other learners

#### Self-assessment of nursing informatics competencies

Scores of students’ nursing informatics attitudes improved significantly after the course. There were no statistically significant changes observed in the scores of students’ competency including the role of clinical informatics, basic computer knowledge and skills, applied computer skills, and wireless device skills (Table 5).

**Table 5:** Self-assessment of nursing informatics competencies pre- and post-course

Factor	Pre- course		Post- course		t	*P*
	Mean	SD	Mean	SD		
Role of clinical informatics	13.08	5.11	13.00	3.32	0.062	0.951
Basic computer knowledge and skills	33.75	7.66	33.54	7.26	0.107	0.916
Applied computer skills	8.38	3.68	9.67	3.29	-1.210	0.239
Wireless device skills	10.46	4.18	11.75	3.03	-1.325	0.198
Nursing informatics attitudes	16.88	2.54	18.46	1.96	2.486	0.021

#### Students’ satisfaction with Digital Health and Informatics course

All 24 Students responded to six questions regarding the effects of the digital health and informatics course (Table 6). A total of 50% of the students indicated that “I am now somewhat familiar with the concept of (nursing) informatics.” and 41.7% of the students indicated that“I am now really familiar with the concept of (nursing) informatics.” Also, a total of 83.3% of the students indicated that,“The learning made me more sensitive to issues related to (nursing) informatics”.

Furthermore, based on the focus group discussions and responses to two open-ended questions in the course evaluation form, three themes were generated including the pros and cons of the course, and suggestions for the course. Details are presented in Table 7.

**Table 6:** Evaluation of Digital Health and Informatics Course

Survey question	Item	Frequency (%)
**After attending the course, to what extent are you now familiar with the concept of nursing informatics? Choose one answer.**		
	A I am still at least a little confused about the concept of nursing informatics.	0
	B I am now somewhat familiar with the concept of nursing informatics.	12(50)
	C I am now really familiar with the concept of nursing informatics.	10(41.67)
	D I am now ready to use nursing informatics related knowledge in my future work.	2(8.3)
	E I feel expert now to start using nursing informatics related knowledge in my future work.	0
**What impact**,** if any**,** will the course have on your ability to use nursing informatics? Choose as many answers as are true for you.**		
	A The learning reinforced my previous thoughts on nursing informatics	16 (66.6)
	B The learning made me more sensitive to issues related to nursing informatics	20(83.3)
	C The learning will likely change how I use nursing Informatics at my future clinical practice	16(66.6)
	D The learning did not do enough to prepare me to deal with work situations related to nursing informatics	4(16.6)
**After attending the course**,** to what extent are you now familiar with the concept of eHealth? Choose one answer.**		
	A I am still at least a little confused about the concept of eHealth.	2(8.3)
	B I am now somewhat familiar with the concept of eHealth.	14(58.3)
	C I am now really familiar with the concept of eHealth.	3(12.5)
	D I am now ready to use eHealth in my future work.	5(20.8)
	E I feel expert now to start using eHealth in my future work.	0
**How motivated are you to learn nursing informatics? Choose one answer.**		
	A I do not need nursing informatics in my future work.	
	B It is not a priority for me to use nursing informatics in my job.	1(4.2)
	C I plan to learn nursing informatics in my study, but it is not a high priority.	5(20.8)
	D It has medium priority for me to start learning nursing informatics.	8(33.3)
	E It has high priority for me to start learning nursing informatics.	8(33.3)
	F It has very high priority for me to (start) learn nursing informatics.	2(8.3)
**To what extent are you now able to use nursing informatics yourself in future clinical practice? Choose one answer that best describes the extent to which you consider yourself capable of doing so.**		
	A I do not need to use nursing informatics in future clinical practice.	0
	B I still don’t know what to do, and/or why I need to do it.	0
	C I need a little more guidance before I know how to use nursing informatics myself.	4(16.6)
	D I need more experience to be able to use nursing informatics properly.	19(79.2)
	E I can now use nursing informatics myself (without guidance or further experience).	0
	F I feel like an expert now and can use nursing informatics with ease.	0
**Which learning activity did you spend the most time on during the course? Please select up to three options.**		
	A Viewing information presented on a screen (example: via PowerPoint).	20(83.3)
	B Reflecting on how I might use nursing informatics.	11(45.8)
	C Discussing how nursing informatics should be used.	16(66.7)
	D Answering quiz-like questions about the nursing informatics.	9(37.5)
	E Previewing courses related to nursing informatics.	6(25)

**Table 7:** Students’ evaluations and suggestions on the course

Themes	Coding of students’ answers	Frequency*
**Pros**		
	- Broad horizons in informatics for future nursing career choice	12
	- Group discussion to practice teamwork skills	12
	- Case analysis on the use of informatics and digital health in the clinical practice	7
	- Timely problem-solving and technical support by teachers	6
	- Clear and informative presentation slides	6
	- A team Project and presentation to review all previous knowledge	5
	- Online resource sharing in the course online platform	5
**Cons**		
	- Many teaching activities focus on basic computer and internet use rather than on specific nursing informatics profession	7
	- A lack of videos of nursing informatics nurses’ work	5
	- The long duration of teaching activities with three hours per class	4
	- Few group discussions	4
	- Old reference of nursing informatics	4
**Suggestions**		
	- Increase detailed information and examples on the roles and functions of nursing informatics nurse	20
	- Increase practice lessons on digital health use	15
	- Increase videos or individual interviews on the real examples of nursing informatics and digital health use in clinical practice	15
	- Increase group discussions and more knowledge on nursing informatics profession	12
	- Upload some test questions to facilitate testing of learning content	5
	- Increase interactive sessions in the course	5
	- Adjust the duration of teaching activities	4

*The numbers indicate the frequency of key words mentioned by 24 students in the course evaluation form and focus group discussions

## Discussion

Digital health and informatics education is an unmet need to address the global shortage of health workers by promoting the adoption of digital health technologies among future HCPs. This study analyzed the impacts of an online digital health and informatics course for nursing students in China. Students reported improved knowledge and comprehension of key digital health and informatics topics, nursing informatics attitudes in the measure of self-assessment of nursing informatics competencies, and high satisfaction with the course. Also, qualitative results showed notable advantages of the course, including the provision of a broader understanding of informatics for future careers, opportunities for engaging in group discussion, and case analysis on the use of informatics and digital health in clinical practice. The findings of this study provide recommendations for the design and implementation of incorporating digital health and informatics education into the course for health professional students.

### What content of digital health and informatics education should be provided?

Previous research has shown that an individual’s performance expectancy has an impact on their acceptance and use of information technologies in the workplace [24]. Our study highlights that improving education on the role of HCPs in health information systems, raising awareness of the impact of informatics, and introducing emerging digital health technologies in clinical care can potentially enhance the adoption of digital health technologies. This finding is consistent with previous research [25].

To ensure that students develop a comprehensive understanding of digital-enabled healthcare, we have designed this course to provide a broad and foundational education in digital health and informatics for undergraduate students. However, we did not observe significant improvements in students’ self-assessment of nursing informatics competencies in the areas of basic computer knowledge and skills, applied computer skills, and wireless device skills. This may be explained by that these domains are more practical aspects of competence in digital health and informatics. Online lectures may not be the most effective approach for developing practical competency, and real-life practice with digital innovation is necessary. Also, nursing students in our study expressed a need for more practice lessons on digital health use. Therefore, we suggest that nursing students should be exposed to specialized digital health innovation use to enhance their digital literacy in clinical decision support and quality improvement in future course development. This could include trainings on handling medical data and applying data in patient care. Also, case analysis should be incorporated to help students understand how technology and data are used in modern health and social care services, such as electronic medical records, telehealth, and mHealth.

### What teaching methods and components should be provided in digital health and informatics education?

Through a review and reflection of this online education, we have identified useful components of the course that can be applied to other educational programs. Firstly, our course utilized information communication technologies to enhance the quality, accessibility, and sustainability of education. Consistent with previous research [26, 27], the online course offers significant benefits in terms of self-paced, self-directed, and personalized learning. Also, due to the adaptability of an online platform, this course can be easily and flexibly implemented in various settings within medical schools with minimal adjustments. Secondly, students reported that a valuable feature of the course is the group work. In our program, students with different levels of ability and readiness in groups worked together in groups to critically reflect on the role of HCPs in informatics and digital health, promoting interactive group work. Students may lack confidence initially. However, through continuous group cooperation, they were able to develop skills and make progress in the learning and building process. Third, given the distance-learning nature of our course, emphasis is placed on online collaboration tools; both formal and informal synchronous and asynchronous communication tools were used to facilitate communications between instructors, students, and members of student groups (e.g., chat rooms, video-conferencing software). Since each course was conducted online, an interactive class management system can provide students with access to course information, reading assignments, and electronic resources for their final presentation.

### How to improve the quality the digital health and informatics education?

Most students are in the first or second year of their bachelor’s studies and have limited experience in the use of computers and wireless devices in clinical practice. Therefore, we recommend that digital health and informatics should be longitudinally integrated into a compulsory course throughout nursing education, with specific learning objectives and content for each year of nursing student education. Previous studies have shown that at the undergraduate level, students should be equipped with the necessary digital skills to practice medicine in a digital-enabled healthcare environment while also assuring the mastery of compassionate care and improving outcomes for patients [28, 29]. Some studies have reported that digital health education should be provided earlier in the medical university curriculum such as in the first year, and that digital health-specific practice or clinical innovation use should be trained in the final year of health professional education [30, 31]. Additionally, prior studies show that specialized digital skills, such as using digital health for specific clinical tasks in an interdisciplinary environment, should be taught in the advanced years of medical training when HCPs enter residency and train to become specialists [32]. Therefore, we suggest that future research should use the digital health competency framework, such as the International Medical Informatics Association Recommendations on Medical Informatics Education, to design and tailor education for the undergraduate health professional students.

Furthermore, we highlighted the value and significance of collaboration efforts between medical school training and clinical practice [33]; using clinical examples to explain more novel digital health applications, such as the application of artificial intelligence or big data in patient-centered care. To ensure that this course is aligned with the technological advances in clinical settings, training to improve digital competencies in students’ clinical practice is necessary. One way to achieve this is by incorporating a practical exercise on the use of digital health technology in the clinical setting, while also considering the need for patient privacy and liability concerns [34].

## Limitations

Nevertheless, several limitations need to be considered. Firstly, the main limitation of the study was that it was a single-arm study. This raises questions about whether the observed improvements in outcomes, such as knowledge, were solely due to the course or if other factors may have influenced the results. For example, students’ previous use of digital health and eHealth literacy levels could have potentially influenced their experience and evaluation of the course. This could have resulted in a bias in their responses towards course evaluation. Additionally, improvements in students’ learning outcomes could be attributed to the test itself, as factors such as participants remembering questions or the questions raising awareness and triggering learning after the pre-test may have influenced the results, independent of our course implementation. Therefore, future course evaluations should consider using a two-arm or Solomon four-group design. Secondly, this was a small study with a sample size of 24 students and may not be generalizable to all health professional students in China or a larger population. Also, study results report the nursing students’ subjectively experienced changes in nursing informatics competence, and no objective measures in this area were conducted. Therefore, self-reported data may be subject to bias, and may not accurately reflect the actual improved competency of the students. Additionally, the measurement of knowledge and comprehension of key digital health and informatics topics must be interpreted with caution since the quiz has not been validated. Furthermore, the duration of the course was only six weeks. The relatively short duration of the course may limit the depth of knowledge and competencies that students can acquire. A longer course with follow-up assessments could provide a more comprehensive understanding of the long-term impacts.

## Conclusions

Digital health and informatics education for future healthcare professionals is an urgent need to equip them to adapt to future digital medical system changes in their workforce. This Online Digital Health and Informatics education showed promising results for undergraduate nursing students in their knowledge and comprehension of the key digital health and informatics topics, nursing informatics attitudes in the self-assessment of nursing informatics competency, and satisfaction. To optimize the digital health course effect, future course developers should improve students’ basic knowledge and comprehension of digital health and informatics. Also, to enable the standard design and scale-up of effective digital health and informatics education for nursing students, collaboration between medical school training and clinical practice is needed to enhance students’ practical exercise on the application of digital health technologies in the clinical setting. We suggest that the content and teaching methods of this course may form a mandatory part of digital health education for health professional students and could be expanded to students in other contexts and countries.

### Electronic supplementary material

Below is the link to the electronic supplementary material.

Supplementary Material 1

Supplementary Material 2

Supplementary Material 3

Supplementary Material 4

Supplementary Material 5

Supplementary Material 6

## Data Availability

The datasets used and/or analysed during the current study are available from the corresponding author upon reasonable request.

## Abbreviations

WHO: World Health Organization
COVID-19: Coronavirus disease
HCPs: healthcare professionals
SANICS: Self-assessment of Nursing Informatics Competencies Scale
