# Supplementary material for: Online digital health and informatics education for undergraduate nursing students in China: impacts and recommendations
Source: BMC Med Educ. 2024 Jul 26;24:803. doi: 10.1186/s12909-024-05785-5 (PMC11282779; doi:10.1186/s12909-024-05785-5)
Supplement: Supplementary file 1 — Supplementary Material 1 [file 12909_2024_5785_MOESM1_ESM.doc]

**Additional file 1 The overview and content of the Digital Health and**

**Informatics course**

#### **Course Overview**

Digital technologies and big data offer tremendous opportunities to improve health care. Given the increasing emphasis on informatics to enhance patient safety and the quality of care, the field of nursing informatics has long advocated for integrating technology to support nursing practices. As a scientific discipline and profession, nursing informatics can facilitate and enhance all aspects of nursing within a medical center, from primary care to advanced levels of treatment. It is an interdisciplinary field that combines cognitive science, computer science, information science, and nursing science and involves the development, analysis, and evaluation of information systems that are supported by technology to enhance and manage patient care. Health Informatics, also known as Healthcare Informatics, is a field that combines computer science, information science, and health science. Its purpose is to assist in the management and processing of data, information, and knowledge to support healthcare and healthcare delivery.

In this course, students are challenged to think about the digital enabled health care of tomorrow. The course includes classes on topics such as digital health, health informatics, nursing Informatics, smart hospital nursing, eHealth, disease self-management, principles of the use of digital health technologies, health care professionals’ roles in informatics.

By the end of the course, participants will be able to:

- Identify trends in digital health and the challenges and transformation opportunities present in the digital health landscape
- Explain the definitions of digital health, health informatics and nursing informatics
- Define the role of digital health technologies such as Electronic Medical Records, Telehealth, mHealth, Cloud Computing play in transforming the digital healthcare industry
- Understand what separates a successful digital health technologies initiative from a failed one
- Identify how digital solutions can support disease self-management
