# Supplementary material for: Online digital health and informatics education for undergraduate nursing students in China: impacts and recommendations
Source: BMC Med Educ. 2024 Jul 26;24:803. doi: 10.1186/s12909-024-05785-5 (PMC11282779; doi:10.1186/s12909-024-05785-5)
Supplement: Supplementary file 2 — Supplementary Material 2 [file 12909_2024_5785_MOESM2_ESM.doc]

**Additional file 2 A quiz to test knowledge and comprehension of key digital health and informatics topics**

**问题1**

**护士们根据哪种说法理解护理信息学被认为是一个专门的实践领域？**

1. 护理信息学研究的重点包括创建标准化护理语言和临床信息数据库。

2. 护士必须成功完成硕士水平的正规教育课程，才有资格参加资格认证考试。

3. 护理信息学作为一个专业领域，其关注的重点是客户、环境、健康和护士。

4. 工作小组和组织必须代表护理信息学的利益。

**问题 2**

**该护士的哪句话表明其具有 “信息素养”？**

1. “我掌握了有效搜索网站并评估其与医疗保健要求相关性的知识”。

2. “我在医疗环境中使用数据库输入患者数据，包括皮肤状况”。

3. “我使用电子邮件与患者沟通，并通过互联网传播信息”。

4. “我能够熟练使用应用软件，如文字处理、电子表格和演示文稿”。

**问题3**

**护士在为患者提供护理时会收集大量数据。哪项是对患者护理有用的高层次 “信息”？**

1.病人的生命体征如下：血压 130/70，心率 88，呼吸频率 24，体温 98.8 华氏度，血氧饱和度 98%。

2.实验室检查结果包括电解质全套检查和全血细胞计数。

3.患者是一名 64 岁的黑人男性，丧偶，因前列腺炎入院。

4.在昨天静脉注射了 1.0 克 Rocephin（头孢曲松钠）后，患者表示今天感觉好多了。

**问题 4**

**哪种说法最能说明信息素养和计算机素养之间的区别？**

1.信息素养在医疗保健中不是必需的，而计算机素养对于计算机医嘱输入是必不可少的。

2.信息素养是使用电子病历的基本技能，而计算机素养则帮助护士获取数据。

3.计算机知识使护士能够推断数据，但不需要信息技能。

4.信息素养是持续学习的基础，而计算机素养是指熟悉个人计算机的使用。

**问题 5**

**哪种说法表明相应护士的信息学能力达到了适当的水平？**

1. 一名信息学专科护士推断数据以制定留置导管护理的最佳实践模式。

2. 新手护士使用电子表格记录用药反应。

3. 一名经验丰富的护士使用 SNOMED 创建数据库。

4. 初级护士利用互联网整合多学科语言。

**问题 6**

**关于推动当今医疗保健服务体系发展的压力，哪种说法是不准确的？**

1. 通过在急症护理环境中实施技术改进，可以消除当前和预计的护士短缺问题。

2. 以证据为基础的实践需要技术的支持，使医疗服务提供者能够利用最新的研究成果。

3. 实施计算机化医嘱输入可减少用药错误和不良事件。

4. 管理式医疗可利用疾病管理来识别慢性病患者，并对其进行有效治疗，以尽量减少并发症和费用。

**问题 7**

**在当前的医疗保健服务系统中，以下哪个因素不是在急症护理环境中实施信息技术的驱动力？**

1.患者安全

2.护士短缺

3.循证实践

4.护理实践中数字原生代人数的增加

**问题 8**

**哪些行为表明护士是在发挥知识工作者的作用？**

1.护士在对腹部手术后八小时的病人进行评估时，听诊到肠鸣音减弱，并将评估记录在电子病历中。

2.上午 11:30 时，客户的血糖为 240，护士根据医嘱为其注射了四单位普通胰岛素，并在电子医疗管理记录中进行了记录。

3.一名术后患者在腹部手术后第二天早上听诊呼吸音减弱，护士鼓励患者每隔一小时而不是每隔两小时翻身、咳嗽和深呼吸一次。

4.护士获取客户的生命体征 BP-120/82、P-112 和 R-32，合计摄入量 1040 毫升和排出量 1100 毫升，并将这些数据记录在电子病历中。

**问题 9**

**使用电子病历（EMR）的缺点包括以下哪项？**

1.不同级别的医疗服务提供者可同时从多个不同地点访问电子病历

2.一开始需要对员工进行密集培训，使其做好使用电子病历的准备

3.由于需要更多时间进行记录活动，因此用于患者护理的时间减少

4.由于空间限制，无法将诊断图像纳入电子病历中

**问题 10**

**护士长希望聘用一名刚从护理学校毕业的新护士。在信息学能力的初级水平上，该护士应该能够做到以下哪项？**

1.在开始工作的六个月内获得信息学护士证书。

2.在电子病历中输入生命体征和出入量数据。

3.利用互联网查看医疗信息技术的发展趋势。

4.使用电子表格创建人员轮换表。

**问题 11**

**在过去十年中，用药错误不断增加。以下哪些应对措施可以支持最大限度减少或消除这些错误的全国性倡议？**

1.每周生成电子医疗管理记录，并与患者的医嘱进行比较。

2.在给患者用药前，将患者的用药记录与患者的身份手镯进行比对。

3.扫描并比对护士、患者识别手环和处方药的条形码。

4.将患者的用药记录条形码与客户的处方药进行比对。

**问题 12**

**以下哪项是监测系统的最佳范例？**

1.将 132/82 的人工血压输入图形表的生命体征部分。

2.自动血压机测得一个 164/104 的读数后关闭。

3.上午 7:30 和 11:30 的血糖读数分别为 4.9和7.2，并输入计算机系统。

4.体温探针与肺动脉导管相连，以持续监测核心体温。

**问题 13**

**以下哪项是决策支持系统的示例？**

1.用于安排客户在大面积腹部手术后接受后续护理的系统。

2.用于登记在急诊医疗机构接受手术的患者的系统。

3.急诊室管理部门用于在确诊后向患者收费的系统。

4.医生根据选定的入院诊断提供诊断测试建议的系统。

**问题 14**

**哪项陈述表明护士根据关键路径正确记录了文件？**

1.护理文件与其他医疗团队成员的文件分开输入。

2.在护理诊断和结果陈述框架内输入护理文件。

3.如果患者的结果与预期不符，则确定差异。

4.在特定时间内，只为特定患者选择一个关键路径。

**问题 15**

**以下哪项是实施计算机化医嘱输入系统的优势？**

1.医生可以选择继续使用传统的订货方法。

2.当系统处理订单时，每个部门都会同时收到通知。

3.医疗服务提供者可获得更多的系统访问权限或响应时间。

4.临床医生乐于接受与工作流程改变有关的新系统。

**问题 16**

**以下哪个术语被定义为根据术语开发指南开发的结构化和受控语言？**

1.标准化术语。

2.医疗保健标准。

3.医疗术语。

4.健康信息标准。

**问题 17**

**使用以下哪种方法可确保数据收集的准确性和有效性？**

1.标准化术语。

2.药片。

3.条形码。

4.手持设备。

**问题 18**

**有效的自我管理包括：**

**1.监测自己状况的能力**

**2.影响一个人的状况的能力**

**3.影响认知、行为和情绪反应的能力**

**以下选项哪个是正确的**

1.只有 1 是正确的

1. 1 和 3 是正确的
2. 1 和 2 是正确的
3. 所有陈述都是正确的

**问题 19**

**分类系统用于对临床会诊的细节进行分类。什么不是分类系统的功能？**

1.对数据进行分组，以确定治疗的成本和结果。

2.捕捉必要的细节，在护理点记录特定项目。

3.向消费者提供有关治疗方案的成本和结果的数据。

4.用于收集和报告健康统计数据。

**问题 20**

**标准术语的有效性和对病人护理的影响已经过科学调查。因此，使用标准术语可以促进以下工作**：

1.支持多种语言。

2.告知消费者。

3.加强计算机扫盲。

4.促进循证实践和决策支持规则。

**问题 21**

**在护理患者的过程中，一名 21 岁的客户向护士倾诉她去年曾做过人工流产，但没有向家人透露这一信息。以下哪个术语最恰当地描述了这种情况？**

1.同意

2.隐私

3.安全

4.保密

**问题22**

**以下哪种密码最能保证信息和系统安全？**

1.StJohns3821

2.p#3J24q7

3.p#5N24p7#hN5

4.p#3J24q7?hN5

**问题 23**

**跟踪用户访问系统的软件可以创造一个（ ），揭示异常活动或信息的不当使用。**

1.良机

2.审计跟踪

3.渠道

4.流程

**问题 24**

**为什么大多数泄密事件都会发生？**

1.恶意行为

2.系统黑客攻击

3.故意

4.粗心大意

**问题25**

**当地一家养老院的一名注册护士助理 (CNA) 登录了该养老院的临床信息系统。CNA 只能查看注册护士当天分配的客户信息。CNA 无法查看任何被指派客户的财务数据。这种情况属于以下哪种情况？**

1.密码保护

2.计算机取证

3.访问级别

4.用户验证

**English translation**

Question 1

## Nurses understand that nursing informatics is considered a specialized field of practice based on which statement?

1. The focus of nursing informatics research includes the creation of standardized nursing language and clinical information databases.

2. Nurses must successfully complete a master's level formal education program before being eligible to take the credentialing exam.

3. Nursing informatics as a specialty area focuses its attention on the client, the environment, health, and the nurse.

4. Work groups and organizations must represent the interests of nursing informatics.

Question 2

**Which statement by the nurse indicates that the nurse is "information literate"?**

1.“I have acquired the knowledge to effectively search websites and assess their relevance to healthcare requirements”.

2.“I use databases in a healthcare setting to enter patient data, including skin conditions”.

3.“I use e-mail to communicate with patients and disseminate information via the Internet”.

4.“I am proficient in the use of software applications such as word processing, spreadsheets and presentations”.

Question 3

## The nurse collects a significant amount of data when providing care to patients. Which of the following is an example of higher-level "information" that is useful in patient care?

1. The client's vital signs are as follows: blood pressure of 130/70, heart rate of 88, respiratory rate of 24, temperature of 98.8 degrees F, and oxygen saturation of 98%.
2. The results of the lab work include an electrolyte panel and a complete blood count with differential.
3. The patient is a 64-year-old black male who is widowed and was admitted with prostatitis.
4. After receiving 1.0 gram of Rocephin (ceftriaxone sodium) intravenously yesterday, the client reported feeling better today.

Question 4

**Which statement best describes the difference between information literacy and computer literacy?**

1. information literacy is not required in healthcare, whereas computer literacy is essential for computerized medical order entry.

2. information literacy is an essential skill for using electronic medical records, while computer literacy helps nurses access data.

3. computer literacy enables nurses to extrapolate data but does not require information skills.

4. information literacy is the basis for continuous learning, whereas computer literacy refers to familiarity with the use of personal computers.

Question 5

**Which statement indicates an appropriate level of informatics competency for the corresponding nurse?**

1. an informatics-specialized nurse extrapolates data to develop a best practice model for indwelling catheter care.

2. a novice nurse uses a spreadsheet to document medication responses.

3. an experienced nurse creates a database using SNOMED.

4. a junior nurse uses the Internet to integrate multidisciplinary language.

Question 6

Which statement is inaccurate regarding the pressures driving today's health care delivery system?

1. current and projected nurse shortages can be eliminated by implementing technological improvements in acute care settings.

2. evidence-based practice needs to be supported by technology that allows healthcare providers to utilize the latest research findings.

3. implementation of computerized physician order entry can reduce medication errors and adverse events.

4. Managed care can use disease management to identify patients with chronic diseases and treat them effectively to minimize complications and costs.

Question 7

**Which of the following is NOT a driver for implementing information technology in the acute care setting in the current health care delivery system?**

1. patient safety

2. nurse shortage

3. evidence-based practice

4. increase in the number of digital natives in nursing practice

Question 8

**Which behaviors indicate that the nurse is functioning as a knowledge worker?**

1. the nurse auscultates a diminished bowel sound during an assessment of a client eight hours after abdominal surgery and documents the assessment in the electronic medical record.

2. at 11:30 a.m., a client's blood glucose is 240. the nurse administers four units of regular insulin as prescribed and documents this in the electronic medical management record.

3. a postoperative client has diminished breath sounds on auscultation the morning after abdominal surgery; the nurse encourages the client to turn, cough, and take deep breaths every hour instead of every two hours.

4. The nurse obtains the client's vital signs BP-120/82, P-112, and R-32, with a combined intake of 1040 mL and an expired volume of 1100 mL, and documents these data in the electronic medical record.

Question 9

**A disadvantage of using electronic medical records (EMRs) include which of the following?**

1. different levels of healthcare providers can access the EMR from multiple different locations at the same time

2. intensive staff training is required at the outset to prepare staff to use EMRs

3. less time is spent on patient care because more time is needed for documentation activities

4. inability to incorporate diagnostic images into the EHR due to space constraints

Question 10

**A nurse manager wishes to hire a new nurse who has just graduated from nursing school. At the entry level of informatics competency, the nurse should be able to do which of the following?**

1. obtain an informatics nurse certificate within six months of starting work.

2. enter vital signs and access data in the electronic medical record.

3. use the Internet to view trends in healthcare information technology.

4. use a spreadsheet to create a staffing rotation schedule.

Question 11

**Medication errors have been increasing over the past decade. Which of the following responses would support a national initiative to minimize or eliminate these errors?**

1. generating electronic medical management records on a weekly basis and comparing them to the patients’ medical orders.

2. compare the patient's medication administration record to the patient's identification bracelet before administering medication.

3. Scan and compare barcodes of nurse and patient identification bracelets and prescription medications.

4. compare the barcode of the patient's medication record with the client's prescription medication.

Question 12

**Which of the following is the best example of a monitoring system?**

1. an artificial blood pressure of 132/82 is entered into the vital signs section of a graphic chart.

2. an automated blood pressure machine takes a reading of 164/104 and then turns off.

3. Blood glucose readings of 4.9 at 7:30 a.m. and 7.2 at 11:30 a.m. are entered into the computer system.

4. A temperature probe was attached to the pulmonary artery catheter for continuous monitoring of core body temperature.

Question 13

**Which of the following is an example of a decision support system (DSS)?**

1. a system used to schedule clients for follow-up care after extensive abdominal surgery.

2. a system used to register patients who undergo surgery in an acute care facility.

3. the system used by emergency room management to bill patients after a diagnosis has been confirmed.

4. the system used by physicians to provide recommendations for diagnostic testing based on selected admission diagnoses.

Question 14

**Which statement indicates that the nurse has correctly documented documentation according to the critical path?**

1. nursing documentation is entered separately from documentation for other healthcare team members.

2. nursing documentation is entered within the framework of the nursing diagnosis and outcome statement.

3. discrepancies are identified if the patient's outcome is not as expected.

4. only one critical path is selected for a specific patient at a given time.

Question 15

**Which of the following is an advantage of implementing a computerized physician order entry system?**

1. physicians have the option of continuing to use traditional ordering methods.

2. every department is notified at the same time when orders are processed by the system.

3. Healthcare providers can gain more access to the system or response time.

4. clinicians are receptive to new systems related to workflow changes.

Question 16

**Which of the following terms is defined as a structured and controlled language developed according to terminology development guidelines?**

1. standardized terminology.

2. healthcare standards.

3. medical terminology.

4. health information standards.

Question 17

**Which of the following methods can be used to ensure the accuracy and validity of data collection?**

**1. standardized terminology.**

2. tablets.

3. bar codes.

4. handheld devices.

Question 18

**Efficacious self-management encompasses:**

1. **the ability to monitor one’s condition**
2. **the ability to influence one’s condition**
3. **the ability to influence cognitive, behavioural and emotional responses**

**Which of the following statement is correct?**

1. Only 1 is correct
2. 1& 3 are correct
3. 1& 2 are correct
4. All statements are correct

Question 19

**A classification system is used to categorize the details of a clinical consultation. What is NOT a function of a classification system?**

1. grouping data to determine the cost and outcomes of treatment.

2. capturing the necessary details to record specific items at the point of care.

3. to provide consumers with data on the costs and outcomes of treatment programs.

4. for collecting and reporting health statistics.

Question 20

**The effectiveness and impact of standardized terminology on patient care has been scientifically investigated. Therefore, the use of standardized terminology can facilitate the following:**

1. support multilingualism.

2. informing consumers.

3. enhance computer literacy.

4. promote evidence-based practice and decision support rules.

Question 21

**While caring for a patient, a 21-year-old patient confides in the nurse that she had an abortion last year but did not disclose this information to her family. Which of the following terms best describes the situation?**

1. consent

2. privacy

3. safety

4. confidential

Question 22

**Which one of the following passwords provides the most information and system security?**

1. StJohns3821
2. p#3J24q7
3. p#5GN24p7#hN5
4. p#3YJ24q7?hN5

Question 23

**Software that tracks a user's access to a system can create a(n) ( ) that reveals unusual activity or improper use of information.**

1. a good opportunity

2.audit trail

3.channel

4.process

Question 24

**Why do most breaches of confidentiality occur?**

1. Malicious behavior
2. System hacking
3. Intent
4. Carelessness

Question 25

**A Certified Nursing Assistant (CNA) at a local nursing home logs into the home's clinical information system.The CNA can only view information about clients assigned by the CNA for the day.The CNA is unable to view the financial data for any assigned clients. Which of the following is true of this situation?**

1. Password protection

2. computer forensics

3. access level

4. user authentication
