# Supplementary material for: Online digital health and informatics education for undergraduate nursing students in China: impacts and recommendations
Source: BMC Med Educ. 2024 Jul 26;24:803. doi: 10.1186/s12909-024-05785-5 (PMC11282779; doi:10.1186/s12909-024-05785-5)
Supplement: Supplementary file 3 — Supplementary Material 3 [file 12909_2024_5785_MOESM3_ESM.doc]

**Additional file 3 Focus group discussions topic lists regarding knowledge and comprehension of key digital health and informatics topics**

1. Could you please provide an introduction to your knowledge of informatics or digital health?

(**Probe**: Definitions or advancements in informatics and digital health, relevant content on public policy, data management and analysis, patient safety and human health, principles involved in informatics or digital health such as patient data security and privacy, current technology tools in nursing informatics/digital health such as smart hospital care and health system building, emerging technologies for e-health solutions)

1. How do you perceive the importance of (nursing) informatics or digital health in clinical care? In what ways? (Please provide examples)

(**Probe**: The impact of digital health on healthcare, the significance of (nursing) informatics or digital health in clinical care, for instance, smart hospital care and health system building)

1. How do you view the role and function of nurses in (nursing) informatics and digital health?
2. Are there any final points you would like to add regarding any aspect of the subject, or anything you believe we have not addressed?
