# Supplementary material for: Online digital health and informatics education for undergraduate nursing students in China: impacts and recommendations
Source: BMC Med Educ. 2024 Jul 26;24:803. doi: 10.1186/s12909-024-05785-5 (PMC11282779; doi:10.1186/s12909-024-05785-5)
Supplement: Supplementary file 4 — Supplementary Material 4 [file 12909_2024_5785_MOESM4_ESM.doc]

**Additional file 4 A validated Chinese version of the Self-assessment of Nursing Informatics Competencies Scale (SANICS)**

亲爱的同学，您好！为了了解同学们的护理信息能力水平，提高护理信息能力，现请您花10分钟的时间填写以下量表，谢谢配合！

对于每个陈述，用数字1-5来表示您当前的能力水平，其中:1=不能胜任，2=部分胜任，3=完全胜任，4=胜任且精通，5=达到专家水平。

| 量表条目 | 不能胜任 | 部分胜任 | 完全胜任 | 胜任且精通 | 达到专家水平 |
| --- | --- | --- | --- | --- | --- |
| 临床信息角色 |  |  |  |  |  |
| 1.作为一名准护士，参与护理信息系统的选择、设计、实施与评价 |  |  |  |  |  |
| 2.向他人普及或推广护理信息系统的知识或技能应用 |  |  |  |  |  |
| 3.在不涉及保密、法律、伦理及安全问题的前提下促进信息的整合和获取 |  |  |  |  |  |
| 4.在应用计算机技术过程中，遇到伦理决策问题时，能寻求可利用的资源。 |  |  |  |  |  |
| 5作为引领者，能将创新概念和信息学概念融入自己的专业领域 |  |  |  |  |  |
| 基本的计算机知识与技能 |  |  |  |  |  |
| 6.应用电子通信设备(如WFi，移动宽带或其他设备)进行信息交流(通过数据上传、下载等方式) |  |  |  |  |  |
| 7.使用互联网查找/下载感兴趣的项目 |  |  |  |  |  |
| 8.运用数据库管理程序制作简单的数据库或表格 |  |  |  |  |  |
| 9.使用数据库应用程序输入和检索信息 |  |  |  |  |  |
| 10.通过计算机网络技术进行在线文献检索 |  |  |  |  |  |
| 11.使用文字处理、演示图形创建幻灯片(如PPT),并用多媒体演示 |  |  |  |  |  |
| 12.使用文件服务器、万维网等网络导航系统 |  |  |  |  |  |
| 13.对电子文件进行复制、删除、更改目录等操作 |  |  |  |  |  |
| 14.使用现有的外部设备(如 VCD、U盘等) |  |  |  |  |  |
| 15.安全使用计算机技术 |  |  |  |  |  |
| 16.操作windows系统(如使用文件管理器、选择可用打印机、访问已安装应用、创建和删除的目录等)计算机技能的应用能力 |  |  |  |  |  |
| 17.识别计算机系统的基本组件(例如PC，工作站的功能) |  |  |  |  |  |
| 18.在应用程序中进行基本的故障排除 |  |  |  |  |  |
| 19.将应用程序用于诊断编码 |  |  |  |  |  |
| 20.使用应用程序开发测试材料 |  |  |  |  |  |
| 无线设备技能 |  |  |  |  |  |
| 21.从临床数据集中提取数据 |  |  |  |  |  |
| 22.访问共享数据集 |  |  |  |  |  |
| 23.使用无线设备(平板电脑或移动电话)查询和下载用于患者安全和优质护理方面的资源。 |  |  |  |  |  |
| 24.使用无线设备(平板电脑或移动电话)输入数据 |  |  |  |  |  |
|  | 不同意 | 不太同意 | 不确定 | 比较同意 | 同意 |
| 护理信息学态度 |  |  |  |  |  |
| 25.认识到“互联网+健康”将变得更加普遍 |  |  |  |  |  |
| 26.认识到计算机只是促进护理工作的工具，并不能完全代替人的功能 |  |  |  |  |  |
| 27.认识到不是只有计算机程序员才能有效地将计算机技术应用于护理工作中 |  |  |  |  |  |
| 28.认识到临床医生的参与对医疗保健系统的设计、选择、实施和评估的重要性 |  |  |  |  |  |
