# Supplementary material for: Online digital health and informatics education for undergraduate nursing students in China: impacts and recommendations
Source: BMC Med Educ. 2024 Jul 26;24:803. doi: 10.1186/s12909-024-05785-5 (PMC11282779; doi:10.1186/s12909-024-05785-5)
Supplement: Supplementary file 5 — Supplementary Material 5 [file 12909_2024_5785_MOESM5_ESM.doc]

**Additional file 5 A performance-focused course evaluation form**

| **Survey question** | **Item** |
| --- | --- |
| **After attending the course, to what extent are you now familiar with the concept of nursing informatics? Choose one answer.** |  |
|  | A I am still at least a little confused about the concept of nursing informatics. |
|  | B I am now somewhat familiar with the concept of nursing informatics. |
|  | C I am now really familiar with the concept of nursing informatics. |
|  | D I am now ready to use nursing informatics related knowledge in my future work. |
|  | E I feel expert now to start using nursing informatics related knowledge in my future work. |
| **What impact, if any, will the course have on your ability to use nursing Informatics? Choose as many answers as are true for you.** |  |
|  | A The learning reinforced my previous thoughts on nursing informatics |
|  | B The learning made me more sensitive to issues related to nursing informatics |
|  | C The learning will likely change how I use nursing Informatics at my future clinical practice |
|  | D The learning did not do enough to prepare me to deal with work situations related to nursing informatics |
| **After attending the course, to what extent are you now familiar with the concept of eHealth? Choose one answer.** |  |
|  | A I am still at least a little confused about the concept of eHealth. |
|  | B I am now somewhat familiar with the concept of eHealth. |
|  | C I am now really familiar with the concept of eHealth. |
|  | D I am now ready to use eHealth in my future work. |
|  | E I feel expert now to start using eHealth in my future work. |
| **How motivated are you to learn nursing informatics? Choose one answer.** |  |
|  | A I do not need nursing informatics in my future work. |
|  | B It is not a priority for me to use nursing informatics in my job. |
|  | C I plan to learn nursing informatics in my study, but it is not a high priority. |
|  | D It has medium priority for me to start learning nursing informatics. |
|  | E It has high priority for me to start learning nursing informatics. |
|  | F It has very high priority for me to (start) learn nursing informatics. |
| **To what extent are you now able to use nursing informatics yourself in future clinical practice? Choose one answer that best describes the extent to which you consider yourself capable of doing so.** |  |
|  | A I do not need to use learn nursing informatics in future clinical practice. |
|  | B I still don't know what to do, and/or why I need to do it. |
|  | C I need a little more guidance before I know how to use nursing informatics myself. |
|  | D I need more experience to be able to use nursing informatics properly. |
|  | E I can now use nursing informatics myself (without guidance or further experience). |
|  | F I feel like an expert now and can use nursing informatics with ease. |
| **Which learning activity did you spend the most time on during the course? Please select up to three options.** |  |
|  | A Viewing information presented on a screen, (example: via PowerPoint) |
|  | B Reflecting on how I might use nursing informatics. |
|  | C Discussing how nursing informatics should be used. |
|  | D Answering quiz-like questions about the nursing informatics. |
|  | E Previewing courses related to nursing informatics . |
| **Open-ended questions** |  |
| - What aspects/lesson components helped you most in learning and understanding the material? | |
| - What could have been done better to make this a more effective learning experience? Remember, your feedback is critical, especially in giving us constructive ideas for improvement. | |
|  |  |
