# Supplementary material for: Online digital health and informatics education for undergraduate nursing students in China: impacts and recommendations
Source: BMC Med Educ. 2024 Jul 26;24:803. doi: 10.1186/s12909-024-05785-5 (PMC11282779; doi:10.1186/s12909-024-05785-5)
Supplement: Supplementary file 6 — Supplementary Material 6 [file 12909_2024_5785_MOESM6_ESM.doc]

**Additional file 6 Data saturation table: Students’ evaluations and suggestions on the course**

| Themes and codes | *Group ID* | | | | | *Total # quotations* |
| --- | --- | --- | --- | --- | --- | --- |
|  | 1 | 2 | 3 | 4 | 5 |  |
| Pros | 9^a^ | 8 | 11 | 12 | 13(S)^b^ | 53 |
| Cons | 5 | 6 | 4 | 4 | 5(S) | 24 |
| Suggestions | 13 | 12 | 15 | 14 | 22(S) | 76 |

^a^The number represents the number of quotations; ^b^S represents the point of data saturation; no new information emerged for that particular theme or code.
